# Anaphylatoxin C5a Regulates 6-Sulfo-LacNAc Dendritic Cell Function in Human through Crosstalk with Toll-Like Receptor-Induced CREB Signaling

**DOI:** 10.3389/fimmu.2017.00818

**Published:** 2017-07-14

**Authors:** Anouk Zaal, Miranda Dieker, Manon Oudenampsen, Annelies W. Turksma, Suzanne N. Lissenberg-Thunnissen, Diana Wouters, S. Marieke van Ham, Anja ten Brinke

**Affiliations:** ^1^Department of Immunopathology, Sanquin Research, Amsterdam, Netherlands; ^2^Landsteiner Laboratory, Academic Medical Centre, University of Amsterdam, Amsterdam, Netherlands

**Keywords:** complement, IL-10, pro-inflammatory cytokines, 6-sulfo LacNAc dendritic cell, human, crosstalk, LPS, R848

## Abstract

Activation of antigen-presenting dendritic cells (DCs) and the complement system are essential early events in the immune defense against invading pathogens. Recently, we and others demonstrated immunological crosstalk between signaling from receptors recognizing complement activation products and PAMPs on DCs. This affects DC effector function, as demonstrated by the finding that C5a prevents induction of pro-inflammatory cytokines by toll-like receptor (TLR) ligands in human monocyte-derived DCs (moDCs). Here, we demonstrate that this regulatory crosstalk is specifically important in 6-sulfo LacNAc dendritic cells (slanDCs), the most pro-inflammatory DC subset found in human. C5aR and TLR signaling show profound interference in the ERK/p38/CREB1 signaling pathways. C5aR signaling accelerates TLR-induced CREB1 phosphorylation both in moDC and slanDC. This is key in the regulatory effect of C5a on pro-inflammatory DC maturation by mediating induction of IL-10, which subsequently inhibits pro-inflammatory cytokine production *via* negative feedback signaling. Importantly, the regulatory effect of C5a affects T-cell immunity by decreasing Th1 and cytotoxic CD8 T-cell responses. The finding that the pro-inflammatory effector function of slanDC can be down modulated by activation products of the complement system highlights the existence of intricate regulatory interactions between various arms of the immune system. Intensive immune monitoring of patients suffering from complement-mediated diseases or patients receiving complement modulating compounds can give more inside in the contribution of complement receptor and TLR crosstalk in APCs in disease.

## Introduction

Immune protection against invading pathogens often requires both activation of the innate complement system and activation of antigen-presenting dendritic cells (DCs) to induce adaptive immunity. The complement activation product 5a (C5a) is a well-known chemo-attractant but has also been implicated in modulation of mouse antigen presenting cell function (1–7). We previously demonstrated that crosstalk between C5a receptor (C5aR) and toll-like receptor (TLR) signaling dampens the pro-inflammatory potential of human monocyte-derived dendritic cells (moDCs) by decreasing the production of IL-6, TNF-α, IL-12, and IL-23 (8). This inhibitory effect of C5a on human moDC only occurred on maturing DCs, as in the absence of a TLR stimulus, C5a promoted production of the pro-inflammatory cytokines IL-6, TNF-α, and IL-12 (8, 9).

The fact that more than 20 C5/C5a modulating compounds are in preclinical development or have already reached clinical trials (10, 11), emphasizing the relevance for further elucidation of regulation of TLR-mediated DC differentiation by complement activation products. Important questions remain. First, it is unclear, which DC subset in human is subject to regulation by C5a. In blood, four DC subsets have been described, being CD1c+ DC (MDC1), CD141+ DC (MDC2), CD123+ DC (pDC), and 6-sulfo LacNAc dendritic cell (slanDC). slanDCs comprise the most pro-inflammatory DC subset described in human and express high levels of C5aR (12, 13). Increased infiltration by slanDCs is observed at the site of inflammation in several chronic diseases, including psoriasis (13, 14), systemic lupus erythematosus (15), arthritis (16), and inflammatory bowel disease (17). In murine models that mimic some of these immune-mediated diseases, treatment with the C5aR antagonist PMX53 improved disease outcome (18, 19). Although known to be mainly pro-inflammatory, slanDCs can also regulate immune cell activation (20). It is not clear how activation of slanDCs is regulated and if slanDCs are susceptible to regulation by C5a.

Second, signal transduction pathways involved in C5aR and TLR crosstalk in human DCs remain to be elucidated. In mice, Akt, PI3K, MAPKs, and NF-κB have been implicated in C5aR and TLR crosstalk in macrophages (1, 2) or DCs (3), but key signaling molecules have not been identified. Understanding the mechanism behind C5aR and TLR crosstalk in human DCs is relevant to determine the potential importance of this crosstalk in auto-immune diseases and upon pathogen invasion. Mice studies reported that C5aR signaling in macrophages diminished the clearance of *Leishmania major* and *Porphyromonas gingivalis* upon infection (1, 21, 22).

In this study, we show that C5a inhibits pro-inflammatory cytokine production in the potent pro-inflammatory slanDC. Acceleration of TLR-induced CREB1 phosphorylation by C5a plays a central role in inhibition of TLR-induced pro-inflammatory cytokine production as it induces IL-10 secretion. Negative feedback signaling by IL-10 is essential for the inhibitory effect of C5a in both slanDC and moDC. The regulatory effect of C5a on moDC pro-inflammatory cytokine production reduces Th1 and cytotoxic T-cell responses, implying that C5a can dampen adaptive immune responses by modulating slanDC function.

## Materials and Methods

### Reagents

Cellgro DC serum-free medium, IL-4, and GM-CSF were obtained from CellGenix (Freiburg, Germany). C5a and Fetal Calf Serum were from Sigma-Aldrich (St. Louis, MO, USA). LPS (lipopolysaccharide, *Escherichia coli* 0111:B4, Ultrapure; TLR4 ligand), and R848 (imidazoquinoline compound; TLR7/8 ligand) were from InVivoGen (San Diego, CA, USA). Penicillin/streptomycin was obtained from Life technologies (Gibco^®^; Carlsbad, CA, USA). BIRB796 was obtained from Selleckchem (Munich, Germany), U0126 was obtained from Calbiochem (Darmstadt, Germany), SB747561A was obtained from Tocris Bioscience (Minneapolis, MO, USA), and anti-IL-10 blocking antibody was obtained from Sanquin Research (clone 10.8, Amsterdam, The Netherlands).

The following kit or antibodies were used to stain immune cells: anti-HLA-DR brilliant ultraviolet (BUV) 395 (cat# 564040), anti-CD3 brilliant violet (BV) 510 (cat# 563109), anti-CD3 phycoerythrin (PE) (cat# 345765), anti-CD11c PE (cat# 347637, RRID:AB_2129929), anti-CD14 allophycocyanin (APC) (cat# 345787), anti-CD19 BV510 (cat# 562947), anti-CD56 BV510 (cat# 563041), anti-CD123 BV650 (cat# 563405), anti-pP38 (pT180/pY182) Alexafluor (AF) 647 (cat# 612595, RRID:AB_399878), and anti-pERK1/2 (ERK1 pT202/pY204; ERK2 pT184/pY186) AF488 (cat# 612592), which were obtained from BD Biosciences (San Jose, CA, USA). Anti-CD1c PE/Cy7 (cat# 331515, RRID:AB_1953227), anti-CD141 BV421 (cat# 344113, RRID:AB_2562956), and anti-C5aR APC (cat# 344310, RRID:AB_11204420) were from BioLegend (San Diego, CA, USA). Anti-CD14 Qdot800 (cat# Q10064, RRID:AB_2556449) and Fixable Near-IR Dead Cell stain Kit (cat# L10119) were obtained from Thermo Fisher Scientific (Life Technologies). Anti-MDC8 fluorescein isothiocyanate (FITC) (cat# 130-093-027, RRID:AB_871581) was from Miltenyi Biotec (Bergisch Gladbach, Germany). Rabbit anti-pCREB1 (pS133) (cat# 9198L) and rabbit anti-p65 (cat# 8242S, RRID:AB_10859369) were from Cell Signaling Technology (Beverly, MA, USA) and the secondary antibodies goat anti-rabbit AF568 (cat# A-11036, RRID:AB_143011) and goat anti-rabbit AF488 (cat# A-11034) were from Molecular Probes (Invitrogen). 4′,6-Diamidino-2-phenylindole (DAPI) used in Imagestream experiments was from Sigma-Aldrich.

### Human DCs

To isolate slanDCs, PBMCs were isolated from buffy coats, obtained from healthy volunteers upon informed consent (Sanquin, Amsterdam, The Netherlands), by density gradient centrifugation on Lymphoprep (Axis-shield, Oslo, Norway). Next, PBMCs were enriched for slanDCs by elutriation with the Beckman-Coulter JE-6B elutriator, followed by slanDC sorting on the Aria II or Aria III Cell-sorter (BD Bioscience) using the slanDC-specific antibody MDC8 FITC in combination with anti-CD14 APC and anti-CD3 PE antibodies. The gating strategy used during sort included removal of CD3- and CD14-positive cells before selecting for MDC8-positive cells (Figure S1 in Supplementary Material). Isolation typically resulted in more than 97% purity of the slanDC. slanDCs were rested overnight in Cellgro DC serum-free medium with Penicillin/Streptomycin (100 U/ml) and 1% FCS at 37°C, 5% CO_2_.

To generate moDCs, monocytes were isolated from fresh apheresis material (Sanquin) of healthy volunteers upon informed consent using ELUTRA™ cell separation system (Gambro, Lakewood, CO, USA). Purity of monocytes was confirmed with flow cytometry, and monocytes were cultured at 20 × 10^6^ cells in 20 ml Cellgro DC serum-free medium supplemented with penicillin/streptomycin (100 U/ml), GM-CSF (1000 IU/ml), and IL-4 (800 IU/ml) for 7 days at 37°C, 5% CO_2_ as described previously (8). After 7 days, moDCs were harvested and rested for 2 h in Cellgro DC serum-free medium supplemented with penicillin/streptomycin (100 U/ml) and 1% FCS at 37°C, 5% CO_2_ prior to stimulation.

Dendritic cells were plated at a concentration of 0.5–1.0 × 10^5^ cells/well for cytokine production or at 2.5–5 × 10^5^ cells/well for analyses of cytokine mRNA expression. For analyses of phosphoproteins, moDCs were plated at a concentration of 5 × 10^5^ cells/well or PBMCs were plated at a concentration of 2 × 10^6^ cells/well.

### Stimulation of DCs

Dendritic cells were stimulated with LPS (50 ng/ml) or R848 (50 µg/ml) either or not together with C5a (10 nM). The chemical inhibitors SB747561 (1 µM), U0126 (2 µM), BIRB796 (0.1 µM), anti-IL-10 blocking antibody (33 µg/ml) or an IgG1 control antibody (anti-Feld1, 33 µg/ml) were added 30 min prior to TLR stimulation when appropriate. Expression of phosphoproteins was assessed after 0–60 min of stimulation. Cytokine mRNA expression was determined after 2–7 h of stimulation. Cytokine production in the supernatants was determined after 5 h or overnight stimulation.

To analyze CREB1 phosphorylation in slanDC, isolated PBMCs were stained with the Fixable Near-IR Dead Cell stain Kit, followed by labeling with anti-CD14 APC, anti-CD19 BV510 and anti-MDC8 FITC antibodies. Anti-CD19 BV510 was added during PBMC staining for analysis of B cells, which is not within the scope of this manuscript. Next, PBMCs were rested for 2 h in Cellgro DC serum-free medium supplemented with Penicillin/Streptomycin (100 U/ml) and 1% FCS at 37°C, 5% CO_2_ and stimulated as described above. Cells were labeled prior to stimulation because the timing of the experiment did not allow for staining after stimulation and prior to fixation.

### C5aR Expression on Human Blood Dendritic Cell Subsets

PBMCs were stained with Fixable Near-IR Dead Cell stain Kit in PBS followed by staining with the antibodies anti-HLA-DR BUV395, anti-CD1c PE/Cy7, anti-CD3 BV510, anti-CD14 Qdot800, anti-CD19 BV510, anti-CD56 BV510, anti-CD123 BV650, anti-CD141 BV421, anti-MDC8 FITC, and anti-C5aR APC in PBS supplemented with 0.5% BSA, 0.01% sodium azide, and 3 mg/ml human gamma globulin. PBMCs were analyzed by flow cytometry (5-laser Fortessa, BD Biosciences).

Prior to spanning-tree progression analysis of density-normalized events (SPADE), dead cells, and CD3+, CD19+, and CD56+ cells were removed from the FCS files to prevent undesired clustering. For initial clustering during SPADE analysis (to generate the tree), expression of HLA-DR, CD11c, CD1c, CD141, slan (MDC8), CD14, and CD123 was used. An adapted script provided by Stephan Schlickeiser (Institute of Medical Immunology, Charité University Medicine, Berlin, Germany) (23) was used to perform the clustering analysis in R (24). The expression data of three independent donors were used to generate 120 clusters. Expression of individual markers in the 120 clusters was visualized using software packages for R and Cytoscape (25) (v2.8.2) and the CytoSPADE plugin. This allows visualization of one parameter in all clusters generated using a color scale representing the fluorescent intensity of the selected marker. The clusters comprising the different human DC subsets were identified based on the expression of a combination of markers. pDCs were selected as CD123+, CD11c−, CD1c−, CD14−, and slan−; slanDCs were CD11c+, CD1c−, CD14−, slan+, and CD123−; MDC1 were CD11c+, CD1c+, CD14−, slan−, and CD123−; and the MDC2 cluster was CD11c+, CD141++, CD1c−, CD14−, and slan−. To determine C5aR expression, the anti-C5aR antibody was included during staining and visualized in the 120 SPADE clusters.

### Real-time Quantitative PCR and ELISA

Monocyte-derived dendritic cells or slanDCs were lysed in peqGold Trifast (Peqlab, Erlangen, Germany). Glycoblue (Invitrogen, Carlsbad, CA, USA) was added as a carrier, and total RNA was extracted according to manufacturer’s instructions (Peqlab). First-strand cDNA was reverse transcribed using random hexamers (Invitrogen) and SuperScript II, RNase reverse transcriptase kit (Invitrogen). Cytokine mRNA expression was determined on StepOnePlus using the Sybr^®^ Green PCR method (Invitrogen). Primer sets were selected to span exon–intron junctions and were ordered from Eurogentec (Seraing, Belgium). mRNA expression was normalized using an internal control, 18S rRNA. Primer sequences can be found in Table S1 in Supplementary Material.

The production of TNF-α, IL-10, and IFN-γ was determined using Compact PeliKine Cytokine ELISA kits according to manufacturer’s instructions (Sanquin Reagents). For the detection of IL-12p40, a combination of two anti-IL-12p40 antibodies was used (clone C11.79 and C8.6, Sanquin Reagents).

### Measurement of NF-κB Activation and Phosphoproteins

Monocyte-derived dendritic cells or PBMCs were fixed for 15 min at 37°C using 3.7% formaldehyde and washed with PBS containing 1% BSA after indicated times. Permeabilization was performed overnight in 90% methanol at −20°C. To measure NF-κB, moDCs were washed and stained using rabbit anti-p65 antibody and goat anti-rabbit AF488. DAPI was added, and moDCs were analyzed on the Amnis Imagestream^®^ Mark II (Millipore, Darmstadt, Germany). NF-κB nuclear translocation was analyzed on double positive cells with the nucleus in focus using the nuclear translocation wizard in the Amnis IDEAS software (Millipore). To measure the phosphorylation of ERK1/2, p38, or CREB1, moDCs or PBMCs were washed and stained with anti-pERK1/2 AF488, anti-pp38 AF647, or rabbit anti-pCREB1, followed by goat anti-rabbit AF488 (moDC) or goat anti-rabbit AF568 (PBMC). Cells were analyzed by flow cytometry (five-laser LSRII, BD Biosciences, different settings were used for PBMC and moDC). slanDCs were gated in the PBMC fraction as CD14 negative and MDC8 positive.

### Co-Culturing DCs and T Cells

CD4+ and CD8+ T cells were isolated from fresh apheresis material (Sanquin) of healthy volunteers upon informed consent using the ELUTRA™ cell separation system (Gambro) and CD4+ and CD8+ isolation kits (Miltenyi Biotec). moDCs were plated in 96-well plates (25,000 DCs; ratio 1:8) and rested at 37°C, 5% CO_2_ for 2 h. moDCs were stimulated overnight with 50 µg/ml LPS in the absence or presence of 10 nM C5a, after which 200,000 CD4+ or CD8+ T cells were added. Co-cultures were incubated at 37°C, 5% CO_2_, and supernatants were collected after 6 days of stimulation.

### Statistical Analysis

Data were analyzed for statistical significance using the GraphPad Prism Version 6.0 software (La Jolla, CA, USA, RRID:SCR_002798). Ratio paired *t* test was used for normalized data. Otherwise, a paired *t* test or one-way ANOVA (when comparing more than two conditions) was used. Results were considered significant when *P* values were below 0.05 (*P value < 0.05, **P value < 0.01, ***P value < 0.001, ****P value < 0.0001). Error bars represent SEM or culture duplicates when representative figures are shown.

## Results

### C5aR Is Almost Exclusively Expressed on slanDCs

**Figure 1:**
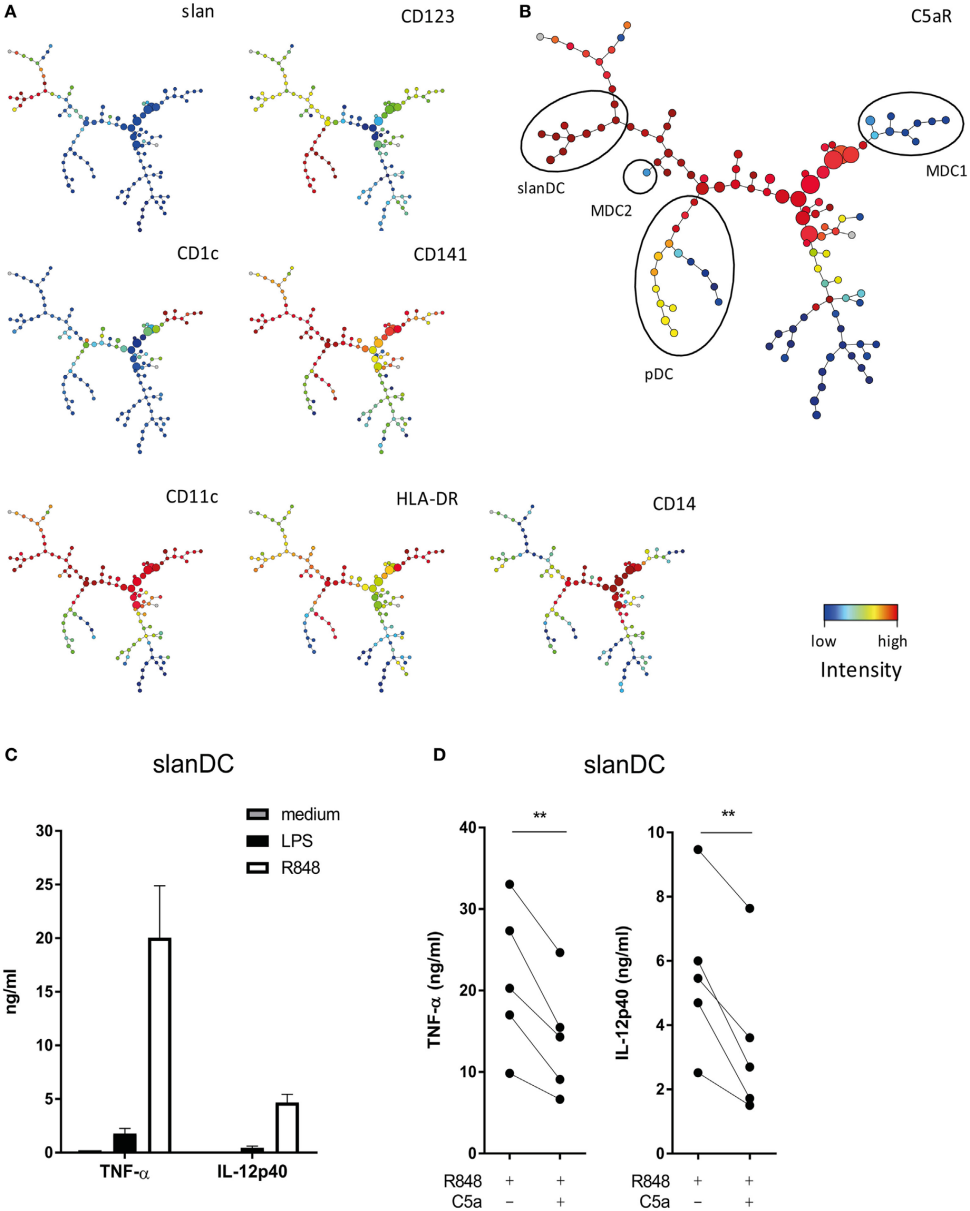
C5a affects the pro-inflammatory potential of 6-sulfo LacNAc dendritic cells (slanDCs). **(A)** Slan, CD123, CD1c, CD141, CD11c, HLA-DR, and CD14 expressions in the 120 different clusters defined by spanning-tree progression analysis of density-normalized events (SPADE). **(B)** C5aR expression in the 120 clusters defined by SPADE. Different dendritic cell (DC) subsets are indicated with black ellipses and were selected based on the expression of known DC identification markers (12, 13). pDCs were selected as CD123+, CD11c−, CD1c−, CD14−, and slan−; slanDCs were CD11c+, CD1c−, CD14−, slan+, and CD123−; MDC1s were CD11c+, CD1c+, CD14−, slan−, and CD123−; and MDC2s were CD11c+, CD141++, CD1c−, CD14−, and slan−. **(A,B)** Fluorescent intensity of marker expression is visualized using a color scale. **(C)** Sorted slanDCs were stimulated overnight with LPS or R848 or left untreated. The production of TNF-α and IL-12p40 in supernatants is depicted (*n* = 4). **(D)** The production of TNF-α and IL-12p40 by slanDC determined after overnight stimulation with R848 in the absence or presence of C5a (*n* = 5). Cytokine production was measured using ELISA.

To investigate which DC subsets in human blood may be especially sensitive to modulation by C5a, expression of C5aR was compared between all four DC subsets present in human blood, being CD1c+ DC (MDC1), CD141+ DC (MDC2), CD123+ DC (pDC), and slanDC, using multiparameter flow cytometry. SPADE (26) was used to visualize expression data into 120 clusters. Clusters comprising the four human blood DC subsets were identified by mapping expression of known DC subset identification markers (Figures 1A,B) (12, 13). Analysis of C5aR expression in these 120 clusters revealed that C5aR was most strongly expressed on monocytes (CD14+ clusters from Figure 1A) and slanDC, whereas no C5aR was found on MDC1 and MDC2 and low-to-intermediate C5aR expression was found on pDC (Figure 1B). These findings are in line with previously reported C5aR expression by human DC subsets (12, 13).

### C5a Inhibits the Pro-inflammatory Potential of slanDCs

The finding that of all DC subsets in human blood, C5aR is most strongly expressed on slanDC, suggests that especially slanDC may be prone to regulation by C5a. The modulatory potential of C5a on TLR-mediated pro-inflammatory cytokine production of *ex vivo* isolated slanDCs was investigated. The TLR7/8 ligand R848 was used because R848 activates slanDC stronger compared to LPS (Figure 1C). C5a inhibited the production of TNF-α and IL-12p40 in R848-stimulated slanDCs (Figure 1D). A similar trend was observed in LPS-stimulated slanDCs, even though cytokine production was much lower (Figure S2A in Supplementary Material). No inhibitory effect of C5a on TLR-induced cytokine production was found in *ex vivo* isolated MDC1 (Figure S3 in Supplementary Material). These results show that C5a specifically regulates the differentiation potential of slanDC.

### C5a Rapidly Induces ERK and p38 Phosphorylation in TLR-Stimulated DCs

**Figure 2:**
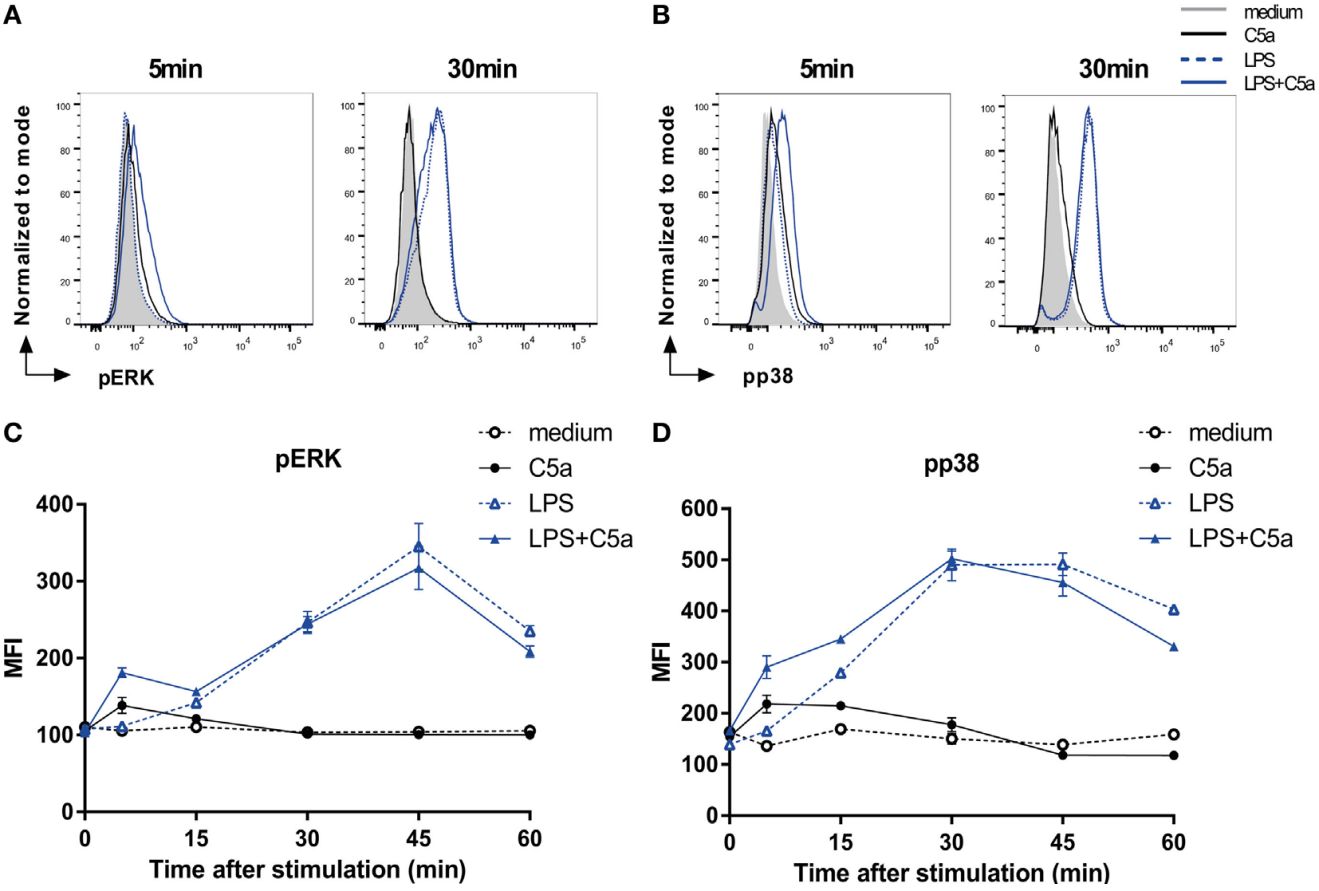
C5a rapidly induces ERK and p38 phosphorylation in LPS-stimulated DCs. **(A)** pERK and **(B)** pp38 expressions in monocyte-derived dendritic cells (moDCs) stimulated for indicated times. **(C,D)** Mean fluorescent intensity (MFI) for **(B)** pERK and **(D)** pp38 in moDCs stimulated in the absence or presence of LPS and/or C5a at different time points. Representatives of six **(A,B)** or three **(C,D)** independent experiments are shown. MFI was determined using flow cytometry. **(C,D)** Error bars represent SEM of duplicate measurements.

To elucidate the mechanism by which C5a downmodulates DC pro-inflammatory cytokine production, potential crosstalk was investigated between known C5aR and TLR signaling cascades involving the phosphorylation of ERK1/2, p38, or JNK or the activation of NF-κB (1, 9, 27–29). Since slanDCs comprise 0.5–2% of the PBMC fraction on average, only low numbers of slanDC could be obtained from each donor (ranging from 0.8 to 3.0 × 10^6^ slanDC per buffy coat). We, therefore, first analyzed potential involvement of specific signaling molecules in human moDCs. Both C5a and LPS-induced ERK1/2 and p38 phosphorylation in moDC (Figure 2), which could be specifically inhibited using MEK1/2 and p38 inhibitors (Figure S4 in Supplementary Material). While C5aR signaling yielded rapid, but limited phosphorylation of both ERK1/2 and p38, TLR4 signaling induced slower, but more sustained ERK1/2 and p38 signaling (Figures 2A–D). Interestingly, C5aR and TLR4 signaling showed cooperation in activation of these signaling molecules, by yielding rapid and prolonged ERK1/2 and p38 phosphorylation (Figures 2C,D). C5a did not affect TLR4-induced nuclear translocation of NF-κB (Figure S5 in Supplementary Material) or JNK phosphorylation (data not shown).

### C5a Accelerates TLR-Induced CREB1 Phosphorylation in Human DCs

To unravel how cooperation of ERK1/2 and p38 activation by C5aR and TLR signaling is conferred onto transcriptional regulation of DC cytokine production, phosphorylation of cAMP responsive element binding protein 1 (CREB1) was assessed. CREB1 forms a common downstream target of both p38 and ERK (30–34) and can bind to the cAMP-response element sequence in the promoter site of various pro- and anti-inflammatory cytokines (35, 36).

**Figure 3:**
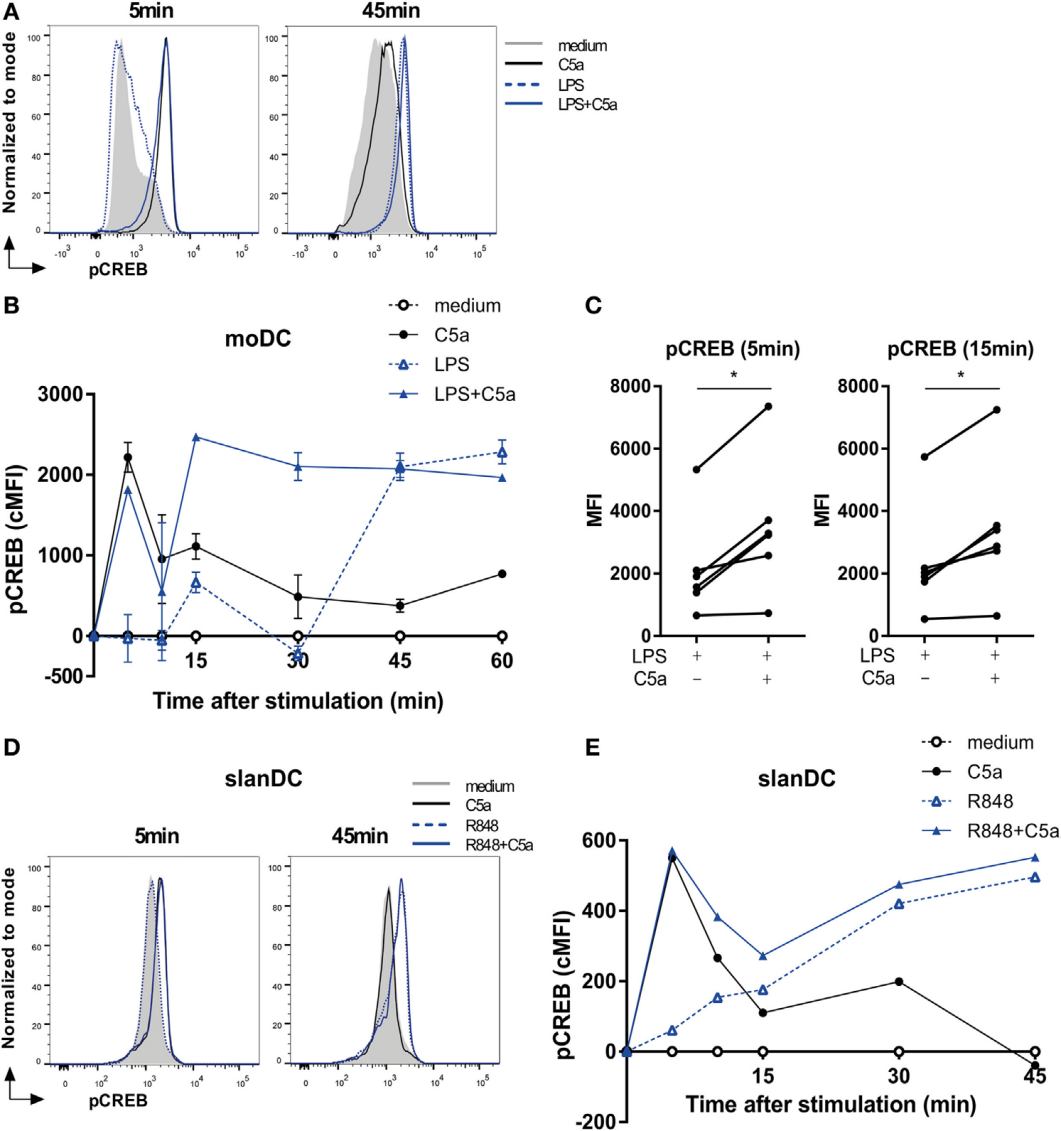
C5a accelerates toll-like receptor-induced CREB1 phosphorylation. **(A)** pCREB1 expression in monocyte-derived dendritic cells (moDCs) stimulated for indicated times. **(B)** Corrected MFI (cMFI) for pCREB1 in moDCs stimulated with or without LPS and/or C5a for different time points. Representatives of **(A)** six or **(B)** five independent experiments are shown. **(C)** Mean fluorescent intensity (MFI) of pCREB1 measured in moDCs stimulated for 5 or 15 min with LPS in the absence or presence of C5a (*n* = 6). **(D)** pCREB1 expression in 6-sulfo LacNAc dendritic cells (slanDCs) stimulated for indicated times. **(E)** cMFI of pCREB1 in slanDCs stimulated in the absence or presence of R848 and C5a for different time points, compared to MFI of unstimulated slanDCs. **(D,E)** Representatives of three independent experiments are shown. MFI was determined using flow cytometry. cMFI was calculated by subtracting MFI of untreated moDCs for each time point.

C5a strongly induced transient CREB1 phosphorylation between 5 and 15 min after moDC stimulation, while LPS-induced a delayed and prolonged CREB1 phosphorylation (Figures 3A,B). Combining DC stimulation *via* C5aR and TLR4 showed that, in addition to the rapid induction CREB1 phosphorylation, C5a strongly accelerated TLR4-induced CREB1 activation at later time points (Figures 3B,C). Investigation of C5aR and TLR7/8 crosstalk in *ex vivo* isolated slanDCs, demonstrated that also here, C5a accelerated TLR-induced CREB1 phosphorylation (Figures 3D,E). Although slanDCs responded much stronger to TLR7/8 stimulation (R848) compared to TLR4 (LPS) stimulation (Figures 1C and 3E; Figure S2B in Supplementary Material), a similar profile was found for LPS-stimulated slanDC (Figure S2B in Supplementary Material).

### C5a Induces IL-10 Production *via* ERK/p38-CREB1 Signaling in TLR-Stimulated DCs

**Figure 4:**
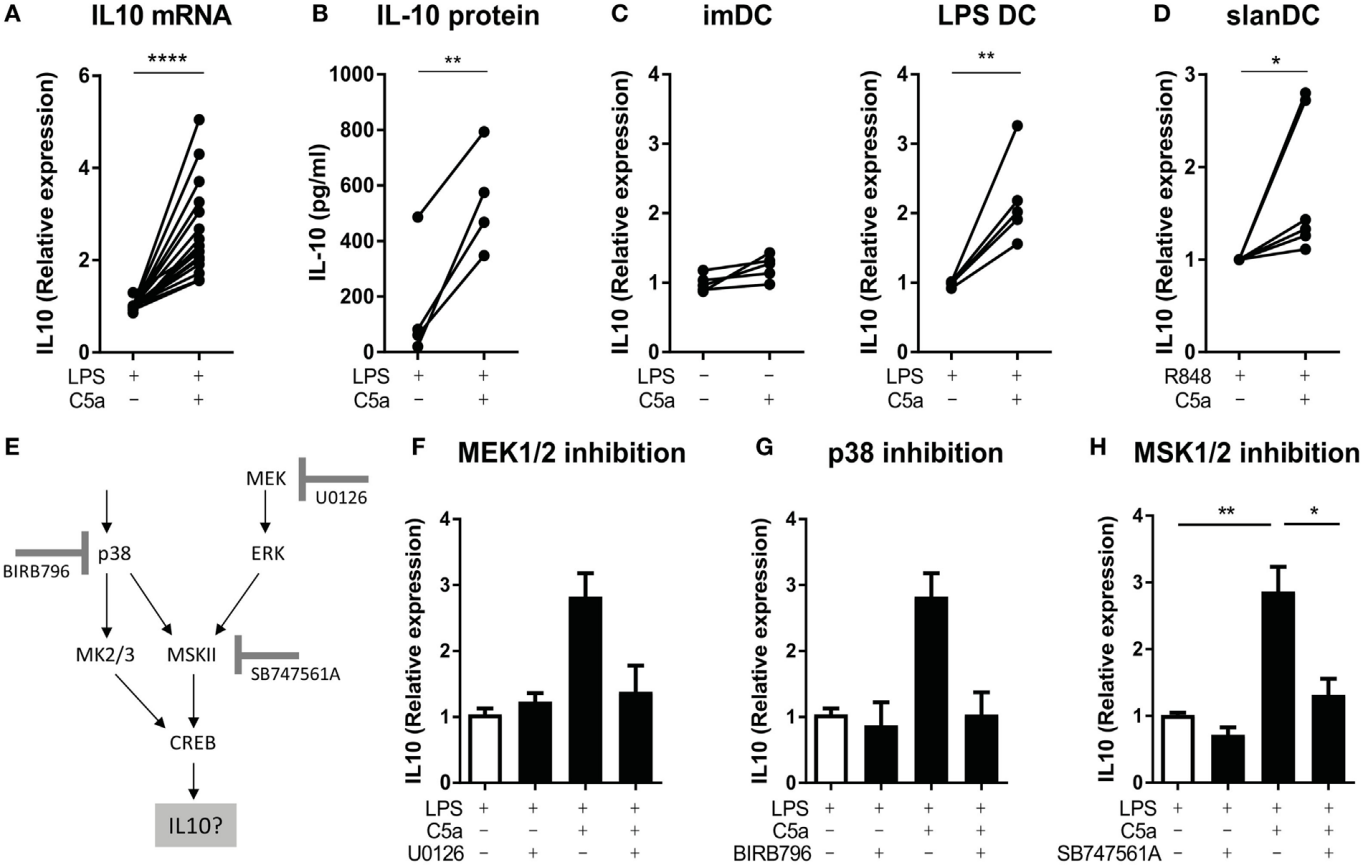
C5a induces IL-10 production in toll-like receptor (TLR)-stimulated DCs *via* p38/ERK-CREB1 signaling. **(A)** IL-10 mRNA expression after 2 h and **(B)** IL-10 production after 5 h of stimulation in monocyte-derived dendritic cells (moDCs) [**(A)**: *n* = 17 and **(B)**: *n* = 4]. **(C)** IL-10 mRNA expression after 5 h of stimulation in unstimulated compared to C5a-stimulated moDCs as well as in LPS-stimulated compared to C5a and LPS-stimulated moDCs (*n* = 5). **(D)** IL-10 mRNA expression in 6-sulfo LacNAc dendritic cells (slanDCs) stimulated for 2 h with R848 in the presence or absence of C5a (*n* = 6). **(E)** Schematic overview on p38/ERK-CREB1 signaling pathway downstream of C5aR and TLR signaling and the three inhibitors used. **(F–H)** IL-10 mRNA expression in LPS-stimulated moDCs after 2 h of stimulation in the absence or presence of C5a and the inhibitor **(F)** U0126 (*n* = 4), **(G)** BIRB796 (*n* = 4), or **(H)** SB747561A (*n* = 10). Relative expression compared to **(C)** unstimulated moDCs, **(A,F–H)** LPS, or **(D)** R848 stimulation is depicted. IL-10 mRNA expression upon LPS stimulation itself is depicted in Figure S6E in Supplementary Material. **(F–H)** Error bars represent SEM of independent experiments.

As the cAMP-response element sequence in the IL-10 gene can bind CREB1 in macrophages (33, 36, 37), we assessed if cooperation between C5aR and TLR in CREB1 phosphorylation induced IL-10 production. C5a significantly increased IL-10 mRNA expression in LPS-stimulated moDC already after 2 h of stimulation (Figure 4A), yielding increased levels of IL-10 protein (Figure 4B). C5a did not induce IL-10 mRNA expression in moDC in absence of LPS (Figure 4C; Figure S6A in Supplementary Material). Analysis of IL-10 mRNA expression in human slanDC demonstrated that also in this very pro-inflammatory human DC subset, C5a increased TLR-induced IL-10 mRNA expression already after 2 h of stimulation (Figure 4D).

To investigate the actual involvement of ERK/p38-CREB1 signal transduction in induction of IL-10 upon C5aR and TLR4 crosstalk, phosphorylation of each of these proteins was prevented using the MEK1/2 inhibitor U0126, p38 inhibitor BIRB796, or MSK1/2 inhibitor SB747561A. Figure 4E illustrates the signal transduction pathway induced downstream of C5aR and TLR4 activation and the inhibitor used. Functionality of the inhibitors and specificity was as expected (Figure S4 in Supplementary Material). Inhibition of ERK1/2 or p38 phosphorylation (Figures 4F,G, respectively), or inhibition of signal transduction downstream of ERK1/2 and p38 and upstream of CREB1 (*via* MSK1/2 inhibition) (Figure 4H) abrogated the ability of C5a to induce IL-10 mRNA in LPS-stimulated moDC. Thus, immunological crosstalk between C5aR and TLR4 signaling at the level of CREB1 signaling is responsible for induction of early IL-10 expression in differentiating human moDC.

### The Effect of C5a on Pro-inflammatory Cytokine Production Is Caused by the Induction of IL-10

**Figure 5:**
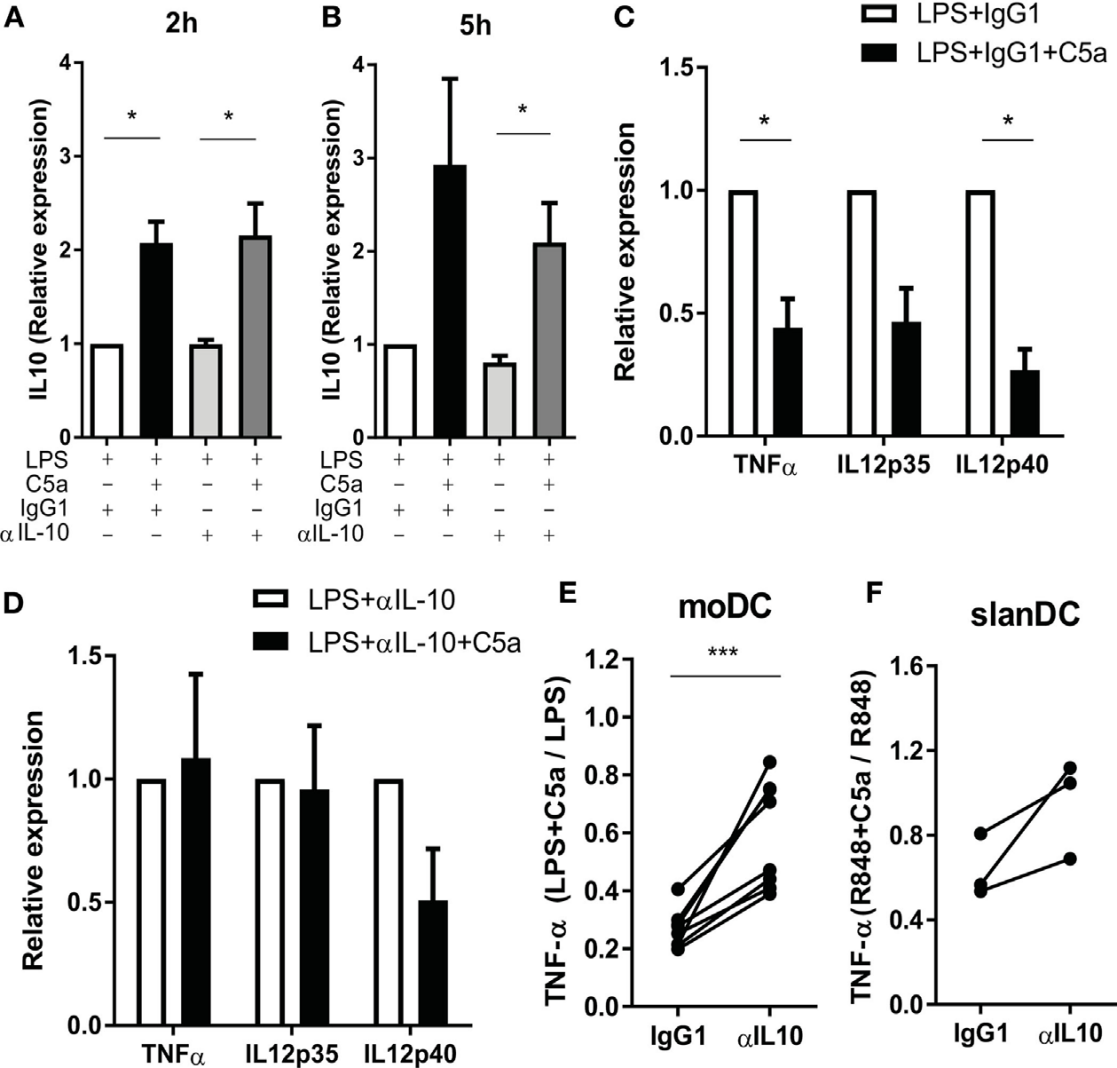
The effect of C5a on pro-inflammatory cytokine production is mainly caused by the negative feedback of C5a-induced IL-10. **(A–D)** Monocyte-derived dendritic cells (moDCs) were stimulated with LPS in the absence or presence of C5a and IgG1 control or IL-10-neutralizing antibody. IL-10 mRNA after **(A)** 2 h and **(B)** 5 h of stimulation. Relative expression compared to LPS-stimulated moDCs is depicted (*n* = 3). **(C–D)** Cytokine mRNA expression after 5 h of stimulation with **(C)** LPS + IgG1 control or **(D)** LPS + anti-IL-10 in the absence or presence of C5a. Relative expression and significance compared to same condition without C5a is depicted (*n* = 3). **(E)** TNF-α production was measured after overnight stimulation of moDCs with LPS in the absence or presence of C5a and an IgG1 control or IL-10-neutralizing antibody using ELISA. Relative expression compared to the same condition without C5a is depicted (*n* = 8). **(F)** TNF-α production after overnight stimulation of 6-sulfo LacNAc dendritic cells with R848 in the absence or presence of C5a and an IgG1 control or IL-10-neutralizing antibody. Relative expression compared to the same condition without C5a is depicted (*n* = 3).

To investigate whether C5a regulates TLR-induced pro-inflammatory cytokine production *via* early IL-10 induction in maturing DCs, IL-10 was blocked during moDC maturation with LPS in presence or absence of C5a. TLR4 stimulation strongly induced mRNA expression of pro-inflammatory cytokines by moDC (Figures S6B–D in Supplementary Material) and the reported inhibitory effect of C5a on LPS-induced pro-inflammatory cytokine production by moDC (8) could be confirmed at mRNA level (Figures S6B–D in Supplementary Material; Figure 5C). Whereas C5a-induced IL-10 mRNA expression was not affected by inhibition of IL-10 (Figures 5A,B), the inhibitory effect of C5a on TNFα and IL-12p35 mRNA expression was fully nullified and the inhibitory effect on IL-12p40 mRNA expression was partially abrogated (Figures 5C,D). The addition of IL-10 blocking antibody during overnight culture diminished the inhibitory effect of C5a on TNF-α production (Figure 5E). In addition, blockage of IL-10 in *ex vivo* isolated slanDCs during C5a and R848 stimulation partially abrogated the effect of C5a on TNF-α production after overnight stimulation (Figure 5F). Thus, the inhibitory effect of C5a on pro-inflammatory cytokine production is mainly dependent on IL-10 induction and subsequent negative feedback of IL-10 in maturing moDC and is at least partly mediated by IL-10 in slanDC.

### C5a Reduces the Capacity of TLR-Stimulated moDCs to Induce Th1 and Cytotoxic T-Cell Responses

**Figure 6:**
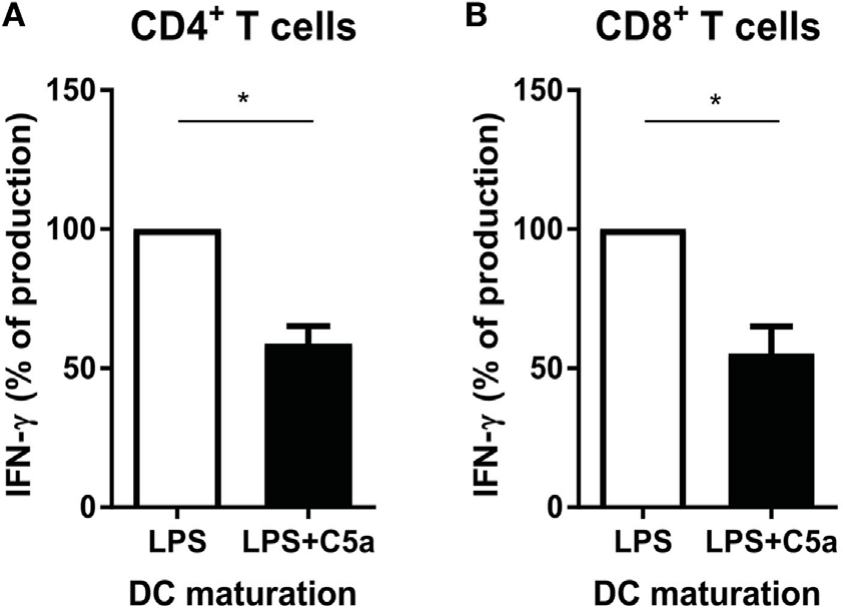
C5a-primed LPS-stimulated DCs reduce Th1 and cytotoxic immune responses. Monocyte-derived dendritic cells were stimulated overnight with LPS or LPS + C5a after which **(A)** CD4+ T cells or **(B)** CD8+ T cells were added (*n* = 3). Supernatants were collected after 6 days of co-culturing. For each independent experiment, the IFN-γ production in co-cultures of LPS-stimulated DCs with T cells (ratio 1:8) was set to 100%.

IL-12 production by DCs is crucial in programming Th1 immune responses. Since C5a inhibited IL-12 production by TLR-stimulated DCs, the effect of C5a on the capacity of TLR-stimulated moDCs to induce Th1 responses was investigated. C5a addition during LPS maturation of moDCs diminished IFN-γ production by CD4+ T cells (Figure 6A). A similar effect of C5a on the capacity of moDCs to induce IFN-γ production by CD8+ T cells was observed (Figure 6B). These findings indicate that C5a reduces the capacity of differentiated moDC to induce Th1 and cytotoxic immune responses.

Taken together, during C5aR and TLR crosstalk, C5a inhibits the pro-inflammatory potential of human moDCs by accelerating TLR-induced ERK/p38-CREB1 signaling, leading to the induction of IL-10 and subsequent negative feedback of IL-10 on pro-inflammatory cytokine production by DCs. Accelerated CREB1 phosphorylation upon C5aR and TLR crosstalk was confirmed in slanDCs, (Figures 3D,E) as well as the involvement of IL-10 negative feedback signaling in the inhibitory effect of C5a on slanDC pro-inflammatory cytokine production (Figure 5F). Furthermore, priming of human moDCs with C5a reduces the capacity of moDC to induce Th1 and cytotoxic immune responses (Figure 6). These data emphasize the widespread functional consequences of complement activation products and demonstrate potential regulation of DC differentiation and subsequent T-cell effector function by complement.

## Discussion

We previously demonstrated that C5a inhibits TLR-induced pro-inflammatory cytokine production in human moDC (8). It remained unclear, however, which human DC subsets present *in vivo* are under the control of C5a upon TLR-mediated maturation. In addition, the mechanism through which C5a conferred its regulation remained elusive. Of all human DC subsets present in blood, C5aR was most strongly expressed by slanDC. Until now, high C5aR expression on slanDCs has only been linked to potent migratory capacity of slanDCs toward C5a (12, 13). In this study, we demonstrated for the first time that C5a regulates the pro-inflammatory potential of slanDC.

Accelerated CREB1 phosphorylation plays a central role during C5aR and TLR crosstalk in human moDCs, as it leads to early induction of IL-10 in maturing DCs, followed by the inhibition of pro-inflammatory DC maturation through negative feedback *via* IL-10. CREB is involved in various cellular processes, including cell proliferation and differentiation, and can interfere with immune responses by modulating NF-κB activity (38). Here, we demonstrate a new function of CREB as being a central regulator of moDC cytokine production, which affected subsequent regulation of T-cell responses during DC encounter with PAMPs. Also, in slanDCs, C5a accelerated TLR-induced CREB phosphorylation and C5a-induced IL-10 production was required for the inhibition of TLR-induced TNF-α production.

Although C5a inhibits pro-inflammatory cytokine production in TLR-stimulated human DC, the opposite has been found in immature DC (8, 9). Since negative feedback *via* IL-10 turned out to be essential for the inhibitory effect of C5a on TLR-induced pro-inflammatory cytokine production, our finding that C5a did not induce IL-10 production in DCs in absence of a TLR stimulus may explain why C5a differently affects cytokine production in immature and mature DCs. Our findings thus show that the effect of C5a on DC effector function depends on the presence of other environmental stimuli, such as PAMPs (9). The induction of IL-10 that we observed in this study seems to contrast with our previous finding showing that C5a did not affect TLR-induced IL-10 production after overnight stimulation (8). Most likely, this difference can be explained by cytokine consumption during the overnight culture, since we did observe differences analyzing IL-10 production at earlier time points.

The important role for IL-10 in mediating the inhibitory effect of C5a on TLR-induced DC cytokine production has not been observed before. In contrast to our results, Seow et al. (27) have described that the inhibitory effect of C5a on TLR-induced TNF-α production was fully IL-10 independent. Their study, however, assessed the effect of C5a on human monocytes and macrophages. Thus, involvement of IL-10 during C5aR and TLR crosstalk seems to be different between cell types. This may be due to differences in IL-10R expression, CREB expression, or IL-10 induction upon stimulation. In line with this, Hutchins et al. (39) found that various LPS-stimulated myeloid immune cells react differently upon IL-10 stimulation and that the expression of proteins involved in IL-10R signaling differs between immune cells. The capacity of IL-10 to interfere with TLR-induced signal transduction pathways may therefore differ between antigen presenting cells.

The inhibitory effect of C5a on IL-12p40 production appeared not to be completely dependent on induction and negative feedback of IL-10. In monocytes and macrophages, C5a inhibits the production of IL-12 in an IL-10-independent manner (1, 40, 41). The presence of both an IL-10-dependent and -independent effect of complement on IL-12 production was also suggested in studies in mice (42). The mechanism behind the IL-10-independent inhibition of IL-12p40 production by C5a needs further investigation. Although implicated to modulate IL-12 production (38), CREB1 signaling is probably not involved here, because preventing CREB1 phosphorylation using the MSK1/2 inhibitor did not diminish the inhibitory effect of C5a on IL-12p40 mRNA expression (data not shown).

Although pro-inflammatory APC functions are required to initiate the appropriate adaptive immune response, regulation of immune responses is necessary to prevent overactive or prolonged immune activation (43, 44). Especially very pro-inflammatory immune cells, like slanDCs, should be tightly regulated to prevent overwhelming adaptive immune responses. Regulation of slanDC by histamine and erythrocytes has been reported (16, 45), but other regulatory mechanisms have not been described. By inducing IL-10 and dampening the production of pro-inflammatory cytokines, C5a regulates TLR signaling in slanDCs, preventing dysregulation which may otherwise lead to overwhelming adaptive immune responses, tissue damage, or IL-12 toxicity (46). In addition, increased production of IL-10 may protect DCs from complement-mediated lysis by inducing expression of several complement regulating factors, such as demonstrated for monocytes and macrophages (47). Regulatory functions of complement have been described before. Truscott et al. (48) demonstrated a strong regulatory effect of complement receptor CD46 in T-cell activation. In macrophages, crosstalk between C5aR and TLR signaling was shown to dampen pro-inflammatory immune responses (1), to suppress clearance of *P. gingivalis* (21), and to induce a switch away from the pro-inflammatory state, while increasing phagocytic capacity (27). Overall, C5a may play an important role in protecting the host by regulating the pro-inflammatory state of slanDCs.

Exposure to C5a during DC maturation affected adaptive immune responses by dampening IFN-γ production by CD4+ and CD8+ T cells. C5a increased IL-10 production by human DCs while dampening the production of TLR-induced IL-12 and TNF-α. This suggests that C5a induces a more tolerogenic phenotype in TLR-stimulated DCs. IL-10 production by DCs has been associated with the formation of regulatory T cells (49, 50), indicating that C5aR and TLR crosstalk may result in an increased induction of specifically this T-cell subset. Although C5aR and TLR crosstalk were previously shown not to affect the expression of co-stimulatory markers by human moDCs (8), the effect of C5a on phenotypical changes typical for DC maturation, as well as the effect of DC on subsequent T-cell differentiation needs further investigation, especially in human slanDCs.

There are several C5/C5a modulating compounds in clinical development to interfere with clinically undesired C5a effector function (10, 11). Although only shown to be successful for the treatment of atypical hemolytic uremic syndrome and paroxysmal nocturnal hemoglobinuria in human, the potential use of these compounds for the treatment of several auto-immune diseases has been investigated not only in murine models for arthritis (18) and inflammatory bowel disease (19) but also in human arthritis patients (51). slanDCs are locally found in high frequencies in both arthritis (16) and inflammatory bowel disease (17) and can produce high levels of several pro-inflammatory cytokines (12, 15, 16). In this article, we found that C5a regulates the pro-inflammatory potential of slanDC by dampening the production of pro-inflammatory cytokines. In addition, Arbore et al. (52) demonstrated that C5a can also directly affect T-cell immunity. The use of C5/C5a modulating compounds in inflammatory diseases may thus not only have desirable effects like damping complement-mediated immunity and influx of immune cells but at the same time may also affect local DC activation and adaptive immune responses. Our findings further emphasize the importance of careful consideration and evaluation of the use of C5/C5a modulating compounds and underscore the importance of understanding the underlying mechanisms and consequences of C5aR and TLR crosstalk in slanDC during disease.

In summary, we demonstrated for the first time that C5aR and TLR crosstalk inhibits the pro-inflammatory potential of slanDC, the most pro-inflammatory DC subset found in human. Acceleration of TLR-induced CREB1 phosphorylation, and subsequent IL-10 induction, is key in the inhibitory effect of C5a on TLR-induced pro-inflammatory cytokine production. C5a priming dampens subsequent Th1 and cytotoxic immune responses induced by moDC. These findings highlight the existence of regulatory feedback mechanisms between two arms of the immune system and emphasize the importance for intensive monitoring upon application of complement modulating compounds.

## Author Contributions

AZ, AtB, and SH designed the research; AZ, MD, MO, and SL-T performed the research and analyzed the data; AT performed the SPADE analysis; AZ made the figures and wrote the manuscript; and AtB, SH, and DW discussed and corrected the manuscript.

## Conflict of Interest Statement

The authors declare that this study received an unrestricted grant from Viropharma. Viropharma was not involved in the study design or collection, analysis, or interpretation of the data.
