# Supplementary material for: Anaphylatoxin C5a Regulates 6-Sulfo-LacNAc Dendritic Cell Function in Human through Crosstalk with Toll-Like Receptor-Induced CREB Signaling
Source: Front Immunol. 2017 Jul 14;8:818. doi: 10.3389/fimmu.2017.00818 (PMC5509794; doi:10.3389/fimmu.2017.00818)
Supplement: Supplementary file 1 [file presentation_1.pdf]

Supplementary Material

ANAPHYLATOXIN C5a REGULATES 6-SULFO-LacNAc DENDRITIC CELL FUNCTION IN HUMAN THROUGH CROSSTALK WITH TLR-INDUCED CREB SIGNALING

Anouk Zaal, Miranda Dieker, Manon Oudenampsen, Annelies W. Turksma, Suzanne N. Lissenberg-Thunnissen, Diana Wouters, S. Marieke van Ham, and Anja ten Brinke*

*** Correspondence:** Anja ten Brinke, a.tenbrinke@sanquin.nl

1 Supplementary Figures and Tables

1.1 Supplementary Figures

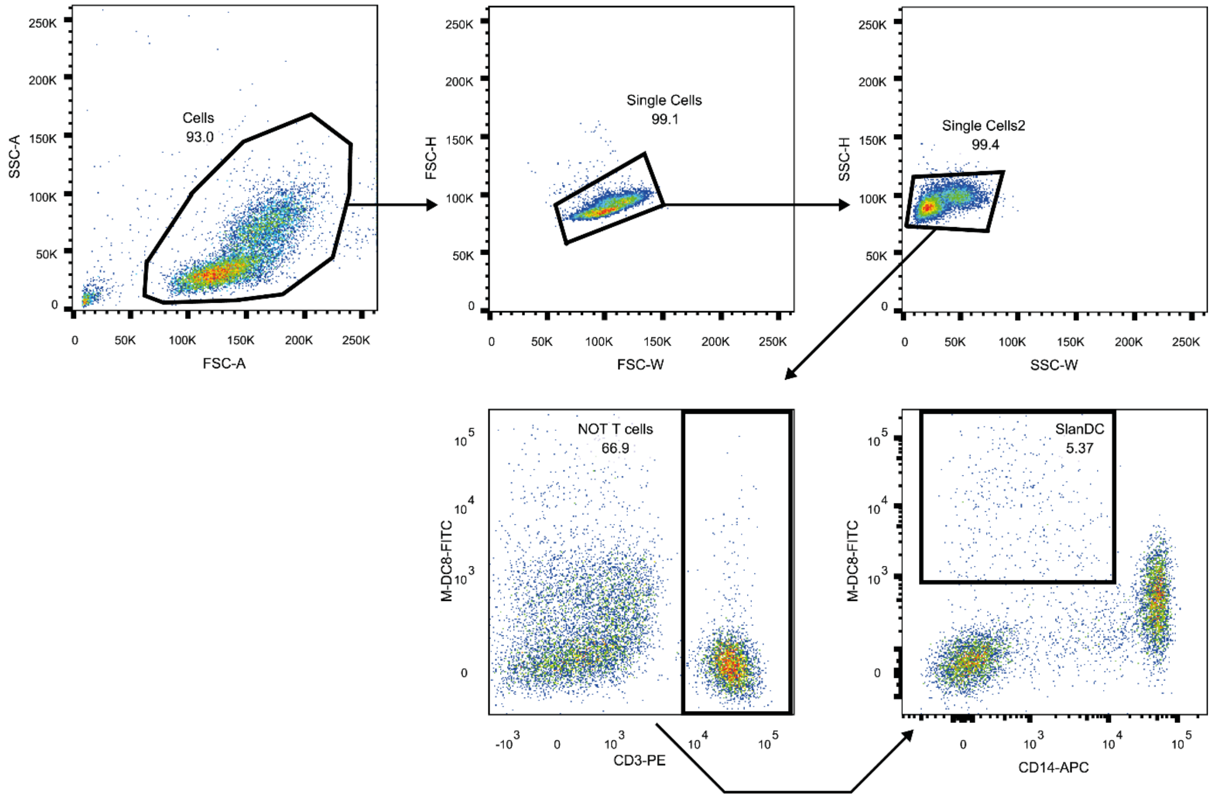

Figure S1. Gating strategy used for slanDC sorting. The enriched PBMC fraction obtained after elutriation was stained with anti-CD3 PE, anti-CD14 APC and MDC8-FITC. slanDCs were sorted by excluding doublets in both the forward scatter and side scatter. Both $CD3^+$ and $CD14^+$ cells were excluded and slanDCs were selected based on MDC8 positivity.

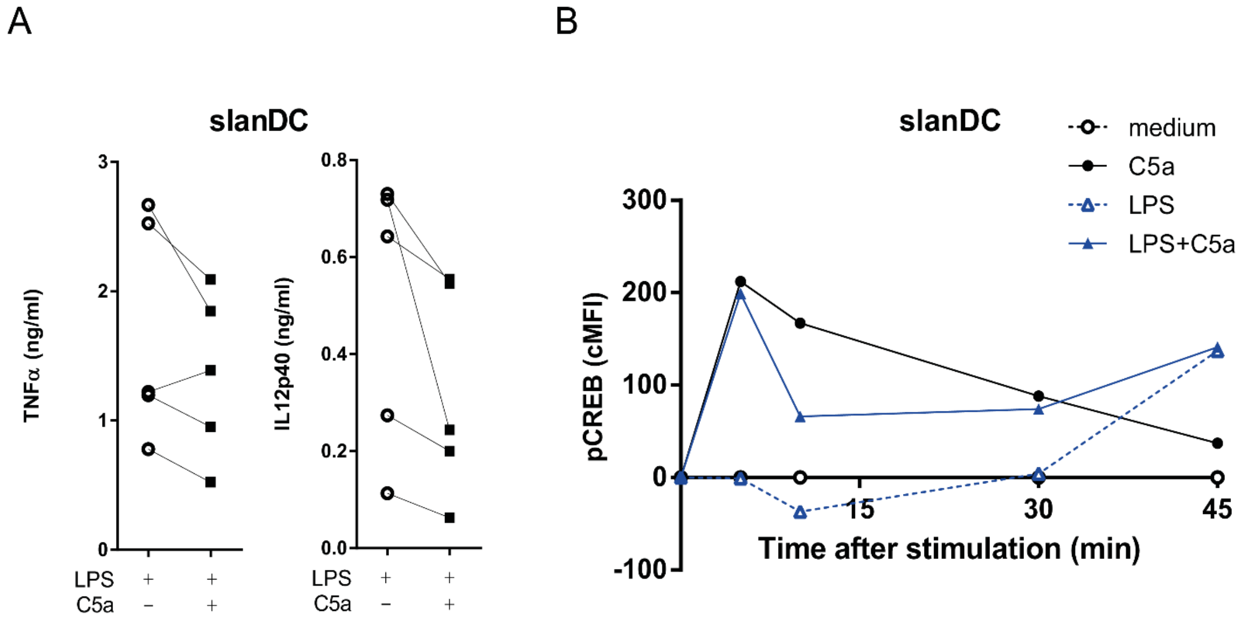

Figure S2. Effect of C5a on LPS-stimulated slanDCs. (A) Sorted slanDCs were stimulated with LPS in the absence or presence of C5a.TNF-α and IL-12p40 production by slanDCs was determined after overnight stimulation using ELISA (n=5). (B) pCREB1 in slanDCs stimulated in the absence or presence of LPS and C5a for different time points. Representative of 3 independent experiments is shown. Intensity was determined using flow cytometry. Corrected MFI was calculated by subtracting MFI of with medium incubated slanDCs for each time point.

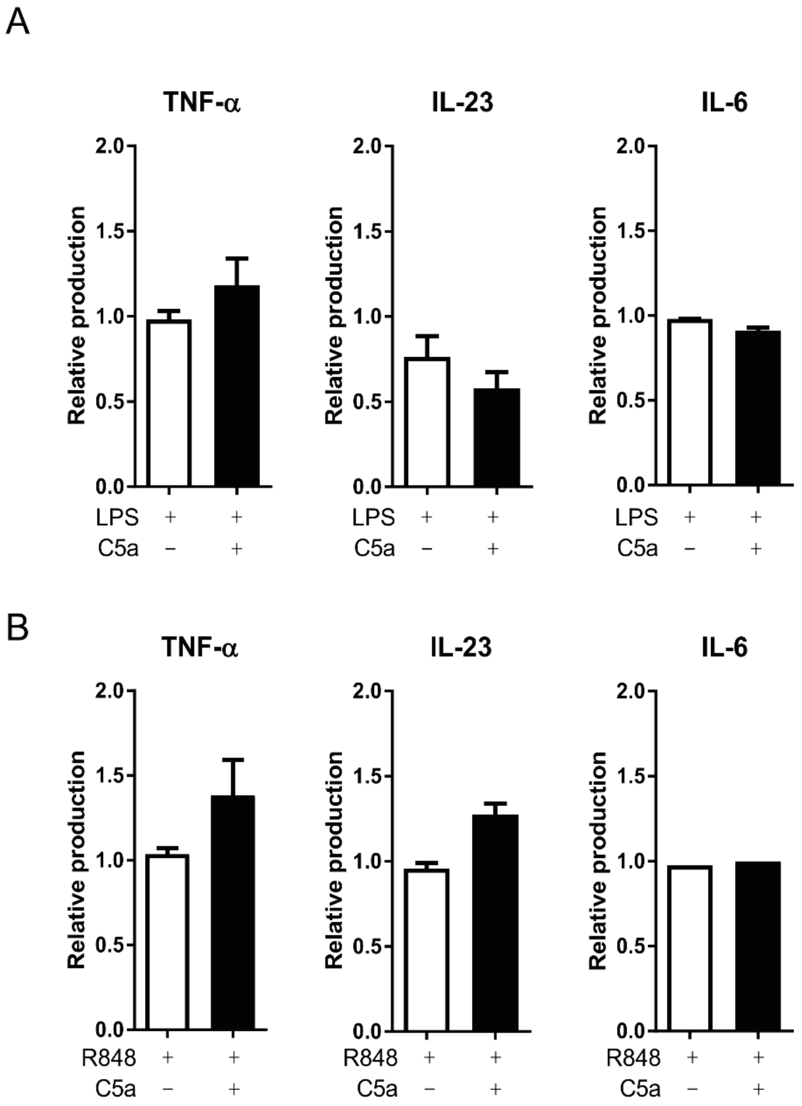

Figure S3. No effect of C5a on TLR-induced proinflammatory cytokine production in MDC1s. MDC1s were isolated using CD1c MACS beads and stimulated overnight with (A) LPS (TNF-α; n=3, IL-23, IL-6; n=2) or (B) R848 (TNF-α, IL-23; n=2, IL-6; n=1) in the presence or absence of C5a. Cytokine production was analysed using ELISA. Expression is depicted relative to stimulation with the TLR ligand.

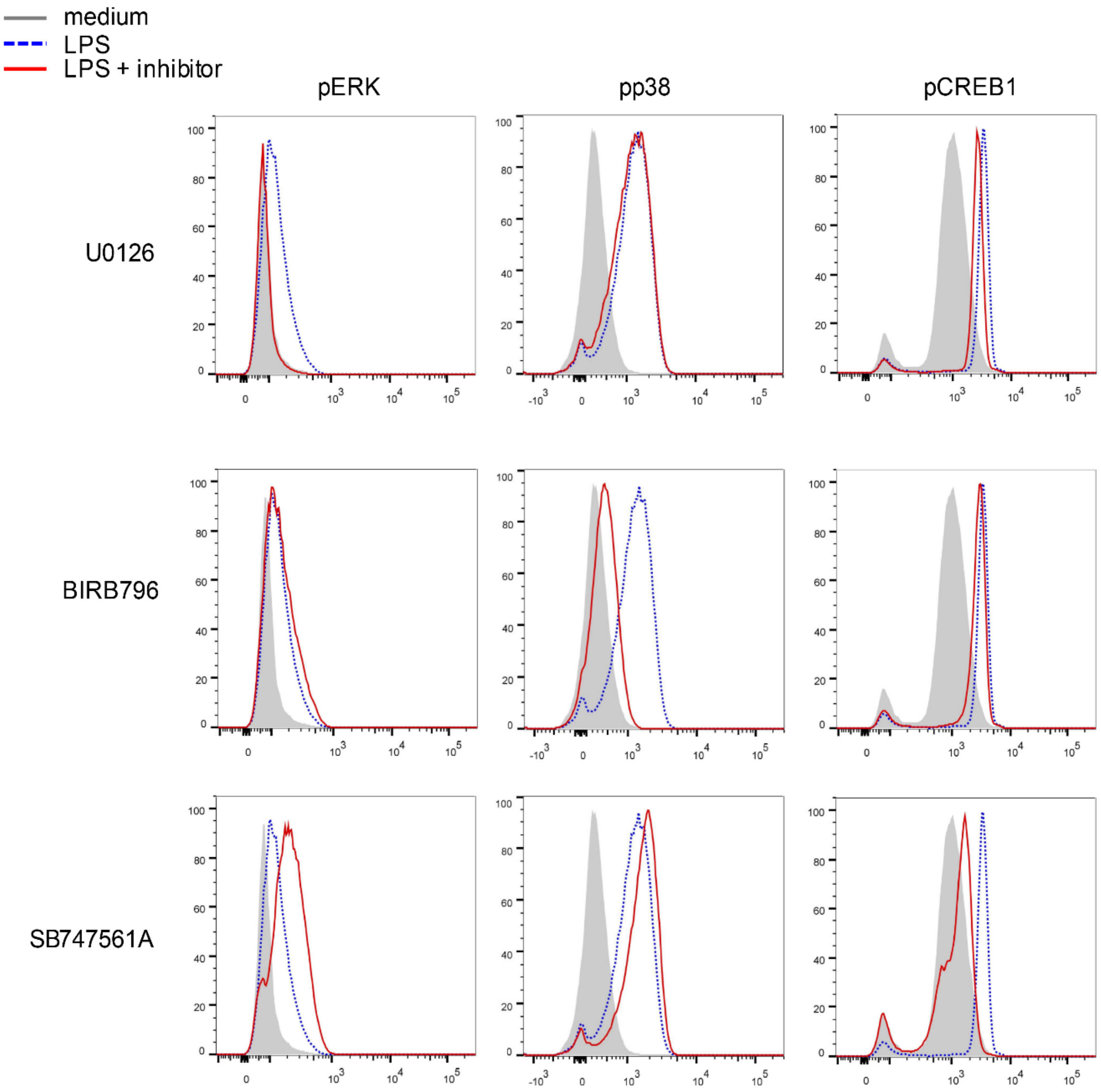

Figure S4. Inhibitors for MEK1/2, p38 and MSK1/2 prevent phosphorylation of ERK, p38 and MSK1/2 in a specific manner. ERK (left), p38 (middle) and CREB (right) phosphorylation in moDCs stimulated with LPS in the absence or presence of the ERK inhibitor U0126 (upper), p38 inhibitor BIRB796 (middle) or MSK1/2 inhibitor SB747561A (lower). Representatives are shown of 2 independent experiments. Intensity was measured using flow cytometry.

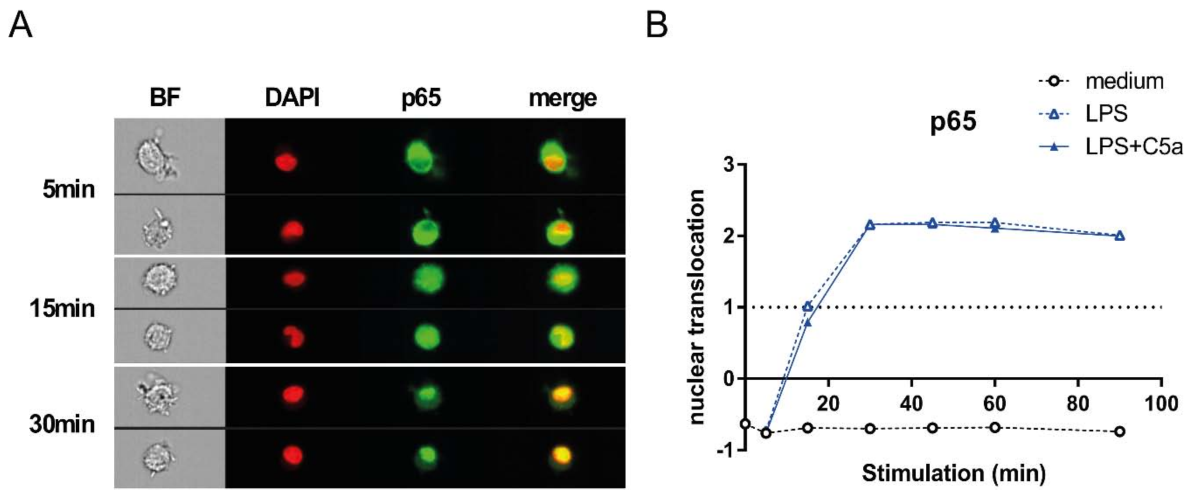

Figure S5. LPS-induced nuclear translocation of NFκB is not affected by C5a. (A) Examples of moDCs stimulated with LPS for 5, 15 and 30 minutes, which corresponds with a mean nuclear translocation of -0.73, 1.0 and 2.2, respectively. (B) Nuclear translocation of p65 in unstimulated moDCs and LPS-stimulated moDCs in the absence or presence of C5a. Representative of four independent experiments is shown.

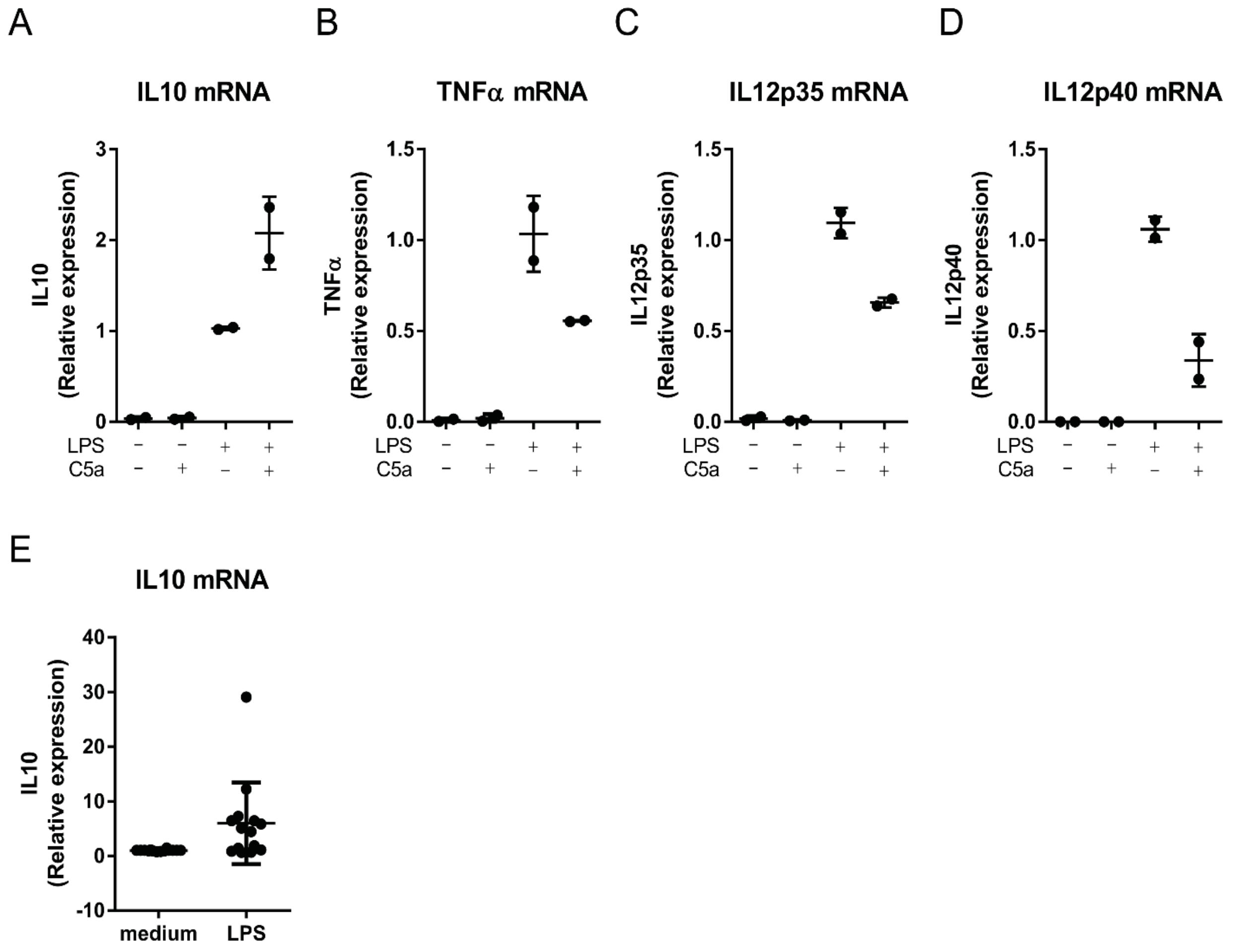

Figure S6. TLR-induced cytokine mRNA expression relative to untreated moDCs. (A-D) Cytokine mRNA expression was determined after five hours of stimulation with C5a, LPS or a combination of the two. mRNA expression was compared to mRNA expression in untreated moDCs to determine TLR induced mRNA induction and subsequent C5a effect. (A) IL-10 mRNA, (B) TNF-α mRNA, (C) IL-12p35 mRNA, and (D) IL-12p40 mRNA expression (n=2). mRNA expression in LPS-stimulated moDCs was set to 1 for one of the two duplicates for each of the independent experiments. (E) IL10 mRNA expression in moDCs stimulated for 2 hours with LPS relative to untreated moDCs (n=14). Error bars represent standard deviation of independent experiments.

1.2 Supplementary Table

Table S1. RTqPCR Primers

Target	Forward	Reverse	Amplicon
TNFα	5'-ACAAGCCTGTAGCCCATGTT-3'	5'- AAAGTAGACCTGCCCAGACT-3'	428bp
IL12p35	5'-TATGAAGACTTGAAGATGTACC-3'	5'-TTGAAATTCAGGGCCTGCATC -3'	137bp
IL12p40	5'-AGACCTTTCTAAGATGCGAGG-3'	5'- CTGCAGAGAGTGTAGCAGC-3'	156bp
IL10	5'-CCTTGTCTGAGATGATCCAG-3'	5'-AAGAAATCGATGACAGCGCC-3'	146bp
18S	5'-CGGCTACCACATCCAAGGAA-3'	5'-GCTGGAATTACCGCGGCT-3'	187bp
